# Virchow

**Published:** 1902-10

**Authors:** 


A Monthly Review of Medicine and Surgery.

EDITOR:

WILLIAM WARREN POTTER, M. D.

All communications, whether of a literary or business nature, books for review and exchanges should be addressed to the editor: 284 Franklin Street, Buffalo, N. Y.

Vol. XLII.—LVIII. OCTOBER, 1902. No. 3.

### Virchow.

THE passing from earth of Professor Virchow which happened September 5, 1902, at his home in Berlin, closed the career of one of the most remarkable medical men that lived during the nineteenth century. So much has been written and published both in the medical and lay press relating to the character and professional work of this great man that nothing new can be evolved here, hence we shall be content to present merely a general review of these tributes gathered from the *New York Tribune* and other sources.

Rudolf Ludwig Karl Virchow was born at Schievelbein, in Pomerania, Prussia, October 13, 1821. When he had reached the age of eighteen years he went to Berlin to study anatomy and physiology at the university. After four years of application he took the degree of doctor of medicine, and was soon appointed to an assistant's place in one of the Berlin hospitals. Here he was placed in charge of the autopsies performed on all who died in the hospital. After three years of this kind of experience Virchow decided to enter on an academic career. He became a privat docent of the Berlin High School, at the same time retaining his hospital position. It was during this period of hospital work that Virchow made important discoveries relative to the white blood-corpuscles.

In 1847, when he was only twenty-six, Virchow founded the *Archiv. für pathologische Anatomie und für klinische Medizin*, a journal which has now passed its 160th volume, and which has been the means of promulgating many discoveries of the highest value to the medical profession. Since 1852 Virchow had sole charge of this publication.

From 1849 to 1856 Virchow held a professorship in the Pathological Academy at Wurzburg. During those years he became interested in the study of anthropology, making what has been described by Dr. Ernst von Bergmann as "the first effective anthropological investigation"—a study of Cretinism, that form of imperfect mental development accompanied by a correspondingly deficient physical development. In later years Virchow made important collections of skulls, implements, weapons, and the like, for anthropological museums. In 1879 he co-operated with Schliemann in the excavations on the site of ancient Troy.

In 1856 Virchow returned to Berlin as professor of pathological anatomy, of general pathology, and of therapeutics. At the same time he became director of the new pathological institute, which had been founded at his instance, and in that position continued until his death. His marvelous industry in the collection of pathological records and microscopical preparations was rewarded by the erection of the splendidly equipped Pathological Museum of Berlin.

He was in many respects a wonderful man. His contributions to scientific literature made his name known and honored in all parts of the world, and despite the fact that he was a hard and industrious worker in his chosen field, he found time to take active part in politics and became there, as in the scientific world, a shining light.

Last October Virchow's eightieth birthday was celebrated by medical men and publicists all over the civilised world. At his own home all classes of the community did him honor. His remarkable physical powers astonished his friends. He spoke at that time for more than two hours, and in a speech to which great men from all parts of the world listened described the development of pathological science.

In his youth he was a "forty-eighter," but did not cease to take interest in public affairs after the revolution, like many of his compatriots. He became a City Councillor of Berlin in 1859, and held the place for forty-two years. In 1862 he was elected a member of the Prussian Chamber, and was a member of that body when he died. He was not an ornamental member, but one of the working body, and as chairman of the committee on finance rendered valuable service. For thirteen years he was a member of the Reichstag, but was displaced in 1893 by a social democrat.

In person Virchow was of less than the average stature and of winning and attractive manners. "His expression," said one of his students recently, "is that of extraordinary courage; his attention once drawn to you, you feel as if he were fixing you in focus on the object glass of his mental microscope." But there was nothing unfriendly in this gaze. Indeed, his manner frequently deserved the adjective "charming," particularly at the dinner table and on social occasions, and few great men have been distinguished by like modesty and unselfishness. His worldwide travel gave him additional breadth of mind and a recognition of the aspirations and achievements of other nations which was quite free from any narrow bias of nationality. When he went to England in 1898 to lecture before the Royal Society of Medicine, his speech was most generously appreciative of the labors and achievements of English scientific men. Indeed, he may be said to have taught the English to value the forgotten name of Glisson, while in Italy he informed the Italians of their debt to Morgagni. For America and American aspirations he always showed great interest, understanding and appreciation, although he never visited this country.

He celebrated his golden wedding on August 25, 1900, and that occasion, like his seventieth and eightieth birthdays, gave his admirers, including all classes from the poor student to royalty, opportunity to show how dear to them and how much they appreciated the "grand old man of Germany."

The entire scientific pathology of today has grown up from the foundation laid by Virchow. In 1858 his work on Cellularpathologie was published and it has become one of the classics of medical literature. It marks the dividing line between ignorance and knowledge. Whatever claims the memory of Virchow may have to perpetuity, and they are many, his first must be in the reconstruction of theories and teachings relating to pathology and the placing of them upon an enduring foundation.

His next great claim to preëminence is the sanitary reforms that he persistently advocated relating to the city of Berlin. For forty-three years he was a conspicuous figure in the municipal council of that city and under his watchful eye it has been changed from a cesspool of filth to the most sanitary city in continental Europe. The water supply, disposal of sewage, general street and house sanitation, hospitals and asylums and everything pertaining to public institutions were all remodeled and renovated at his instigation and in many instances under his special supervision. Berlin, once an unhealthy, disease-breeding capital, is now a model of sanitary perfection and one of the healthiest of the great cities of the world. This transformation was due to the energy, skill and untiring perseverance of Virchow. His pupil, Ernest Wende, of Buffalo, for ten years pursued a similar course in this city with correspondingly proportionate results.

In paying tribute to his memory the *New York Tribune* of September 6, 1902, the day following Virchow's death, among other things said editorially:

In this event the world is greatly bereaved. It has lost one of the greatest intellects and one of the noblest characters of the age. It has lost one of the greatest benefactors of the human race, of this age and of all ages. Men have called Virchow the founder of modern medical science. That characterisation in the singular number may be extreme. At least he was one of its chief founders. He was second to no other, and we count upon the fingers of the hand the few who are worthy to be ranked with him. It was one of the chief glories of the glorious nineteenth century that it practically created the science of healing. To it belonged the epoch-making achievements of vaccination, anesthesia, bacteriology, cellular pathology and antisepsis. Under those five heads we must place nine-tenths of our pathology, prophylaxis and therapeutics and indeed of all medical and surgical science since Harvey. Of the authors of those five achievements one, the inventor of anesthesia, remains undetermined, or disputed. One, the illustrious apostle of antisepsis, still lives in honored and active age. One, who could claim with Pasteur the distinction of primus inter pares in that distinguished company, has just passed away.

The world is the poorer for the removal of so inspiring a personality. It is immeasurably the richer for the results of his works and researches, which cannot be removed from it; and those works were many and varied. We think of him first as the discoverer and demonstrator of cellular pathology, the principle which forms the cornerstone of medical science. That was his chief gift to the whole human race. But it is worthy of remembrance that he was, too, the statesman who organised the financial system of the Prussian Government. He was the municipal sanitarian who gave to Berlin its superb water supply and its unrivaled system of sewage disposal, and thus transformed one of the most unhealthful of European capitals into one of the most healthful. For thirteen years a leader in the imperial parliament, for forty years a leader in the Prussian parliament, and for more than forty-two years a dominant member of the municipal council of Berlin, he presented such an example of the man of thought and the man of affairs in one as the world has seldom seen. More nearly perhaps, than any other man of the age he approximated to the distinction of universal genius. He was for two generations a universal benefactor of the race. His name will in all time be held in universal honor, affection and reverence.

